# The educational community and its knowledge and perceptions of native and invasive alien species

**DOI:** 10.1038/s41598-021-00683-y

**Published:** 2021-11-02

**Authors:** Alejandro J. Sosa, Nadia L. Jiménez, Ana C. Faltlhauser, Tomás Righetti, Fernando Mc Kay, Octavio A. Bruzzone, Iris Stiers, Adriana Fernández Souto

**Affiliations:** 1 Fundación para el Estudio de Especies Invasivas (FuEDEI), Simón Bolívar 1559, (B1686EFA), Hurlingham, Buenos Aires, Argentina; 2 Consejo Nacional de Investigaciones Científicas y Técnicas (CONICET), Godoy Cruz 2290, (C1425FQB), Ciudad Autónoma de Buenos Aires, Argentina; 3 Agroecology, Environment and Systems Group, Instituto de Investigaciones Forestales y Agropecuarias de Bariloche (IFAB), INTA-CONICET, Modesta Victoria 4450, CC 277, (R8400XAC), San Carlos de Bariloche, Río Negro Argentina; 4 Multidisciplinary Institute for Teacher Education (Science & Technology, MILO), Vrije Universiteit Brussel, Pleinlaan 9, (1050), Brussels, Belgium; 5 Instituto de Educación, Universidad Nacional de Hurlingham (UNAHUR), Teniente Origone 151, (B1688AAA), Hurlingham, Buenos Aires, Argentina

**Keywords:** Environmental sciences, Environmental social sciences

## Abstract

Environmental education seeks to foster an appreciation for nature and the impact of humans on it while introducing citizens to scientific thinking. Biological invasions affect different aspects of life on earth and mandate urgent management actions. Education and public awareness are strongly recommended for successful prevention and management of invasive alien species (IAS). This work presents a study on knowledge and perception of the educational community of Argentina about native species and IAS. We designed an on-line semi-structured questionnaire to examine perception of the environment, recognition of native species and IAS and awareness about biological invasions. Educators recognised an important number of biotic components, mostly represented by trees, birds and mammals. Recognition of native species and IAS, and awareness of biological invasions were different between NST (Natural Science Teachers) and non-NST. Respondents had different performances when they were exposed to recognising native species though written names or photographs. Out of 532 respondents, 56% knew what biological invasions are, 21% answered “Maybe” and 23% had never heard about them. We need to foster capacity-building and encourage a two-way communication between educators and scientists, formally and informally, to engage the participation of the whole society in recognition, prevention and management of IAS.

## Introduction

Raising public awareness is important to preserve ecosystems, counteract loss of biodiversity and understand the impact of humans on nature^1–4^. Natural ecosystems provide services that human society needs (i.e., food, medicine, climate regulation, water and soil cycles, pollination, and pest control)^1^. The responsible use of these ecosystem services necessarily requires public understanding. Environmental education, which seeks to foster an appreciation for nature and the impact of humans on it while introducing citizens to scientific thinking, plays an important role in this concern.

One of the main threats that affect ecosystems are biological invasions^5–7^. Invasive alien species (IAS) are moved far from their native ranges into new regions where they can overcome different biogeographical and ecological barriers, with escalating impacts on the environment, the economies and social activities^6–10^. According to Pysek and Richardson^6^ IAS damage ecosystem services, disrupt human well-being and are the 2nd cause of biodiversity decline, therefore IAS mandate urgent actions in the form of prevention, early detection, eradication, management and control activities^11^. While public awareness and education are often considered an important part of prevention they should also be seen as essential to other phases of management of IAS^12^. Since public opinions and attitudes can potentially affect continued introductions and management of IAS, it is imperative to understand the public’s level of knowledge and attitudes toward these pests. Recently, research on biological invasions has recognized the importance of social perceptions of IAS^3,13–16^ with the majority of studies focusing on the general public^17^.

The United Nations Development Programme (UNDP) proclaimed The Sustainable Development Goals^18^ that established the implementation of measures to prevent the introduction and significantly reduce the impact of IAS. In reference to environmental education, it is expected that all learners acquire the knowledge and skills needed to promote sustainable development. To achieve these goals, countries need to substantially increase the number of trained teachers and include the concept of biological invasions or its discipline in education, whether in formal or informal contexts. For this, it is expected that researchers and educators develop, implement and evaluate novel and user-friendly resources and tools^3,19^.

Despite current legislation and actions led by government agencies and non-governmental environmental organizations (NGEO) to raise public awareness, many IAS and native species are still not recognised as such by citizens^16,20^. The topic of biological invasions is relatively new and it has only recently become relevant to environmental educators^4^. Several studies have evaluated the perception of students (children and teenagers) and student teachers^4,7,9,10,21,22^, about biodiversity and IAS in countries such Argentina, Brazil, USA, Germany, Switzerland and Spain. While Gayford^2^ studied perception and understanding of student from their teachers perspective in UK, no studies have analysed such perception in teachers yet.

Teachers as environmental educators are supposed to act as mediators between scientists and students. Moreover, their personal and professional perception of the environment and IAS does have an impact on how teachers approach their tasks as mediators on student learning. In this context, we analysed the perception and knowledge of native species and IAS of the educational community (mostly teachers) of Argentina, with a special focus on those with an environmental or natural science background (Natural Science Teachers, hereafter NST). We (1) explored the perception of the environment, its biotic components, and the global and local threats, of the educational community. We also (2) evaluated the recognition of photographs and written names of native species depending on the NTS condition and respondents age. Finally, (3) we studied the educational community awareness of biological invasions.

## Results

### Characterisation of the respondent

**Figure 1:**
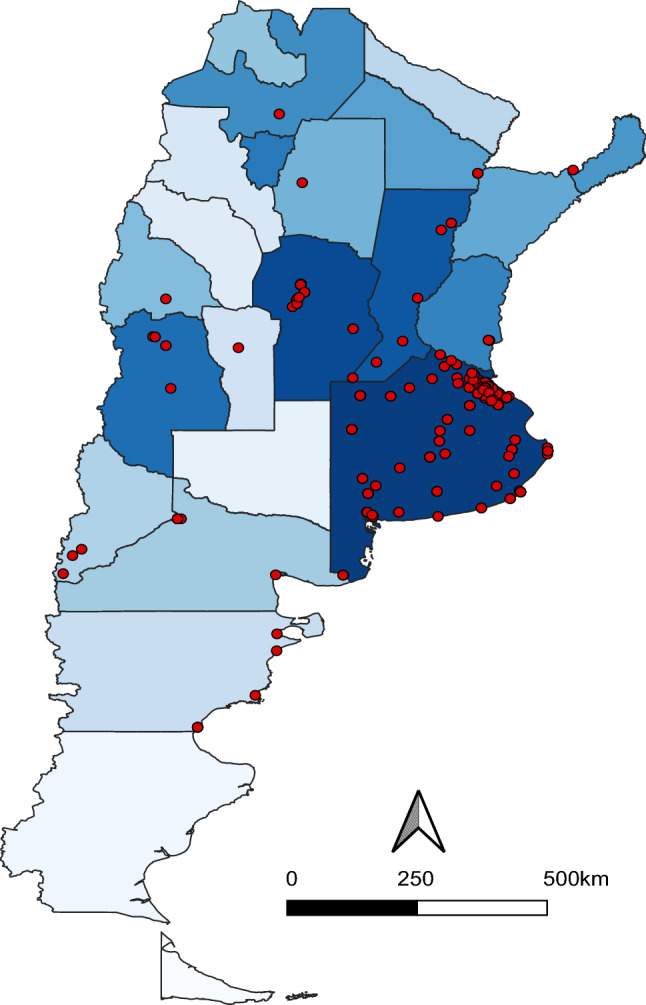
Geographic distribution of the questionnaire respondents (red dots). Blue gradient represents the number of inhabitants per province (state). Dark blue represents a higher number meanwhile light blue represents a lower number of inhabitants. Map was created in QGIS version 3.16 https://www.qgis.org/es/site/.

The internet-based survey questionnaire was completed by 532 respondents, a representative sample of the teacher population of Argentina (Supplementary Appendix S2, Supplementary Eq S1). They were widely distributed in the country, 16 out of 24 provinces (including Buenos Aires city) (Fig. 1). More than half of the responses (279/532) were recorded within the first 30 days. Respondents covered a wide range of ages (mean = 41.21 years old, median = 40.00 years old, Supplementary Appendix S2, Supplementary Table S1, Supplementary Fig. S1).

The educational community was composed by 84% of teachers in activity, 12% student teachers, 3% retired teachers and 1% non-formal education teachers (Table 1). Respondents had been working on education an average of 12.89 years (Supplementary Appendix S2, Supplementary Table S1), 44% in secondary schools (12–18-year-old children), 24% in universities, 19% in primary schools (6–12-year-old children), 9% in Kindergarten (children under 6 years old), 3% in special education, and 2% in non-formal education (and others) (Table 1). Among these, NST comprised 56% while non-NST were 44%.

**Table 1:** Socio-demographic characterisation of respondents.

Characterisation of respondent	Categories	Percentage of respondents (n)
Q3. Currently in teaching employment	Teacher	84 (447)
	Student teacher	12 (64)
	Retired	3 (16)
	Others (non-formal)	1 (5)
Q4. Education level	Complete University	56.8 (302)
	Incomplete University	19.2 (102)
	Complete Postgraduate University	13.5 (72)
	Incomplete Postgraduate University	7.7 (41)
	Complete Secondary School	2.6 (14)
	Incomplete Secondary School	0.2 (1)
Q6. Teaching areas^a^	Secondary School	44.0 (372)
	University	23.8 (201)
	Primary School	18.8 (159)
	Kindergarten	8.8 (74)
	Special Education	2.7 (23)
	Other	1.7 (15)
Q7. Subject	Natural Science Teacher	55.5 (295)
	Non-Natural Science Teacher	44.5 (237)
Q8. NGEO membership	No	85 (452)
	Yes	15 (80)
Q9. Protected area frequency of visiting	> every 3 years	65.4 (348)
	Once a year	26.7 (142)
	Weekly/monthly	7.9 (42)
Q10. Recreational outdoor activities	Walking	45 (239)
	Gardening/Horticulture	26 (138)
	Nature watcher (e.g., birdwatchers)	17 (90)
	Any kind of sport	9 (48)
	None	3 (17)

^a^Some respondents teach in different areas, thus total number of answers (844) is different to the total respondent numbers (532).

Only 15% of the respondents were members of an NGEO, and more than half (65%) visit natural protected areas more frequently than every 3 years. Most respondents (97%) practice some outdoor activity: 45% walk, 26% work in their garden, 17% actively look for animals/plants (e.g., bird watching), 9% sports like hiking or canoeing (Table 1).

### Perception of the environment

**Figure 2:**
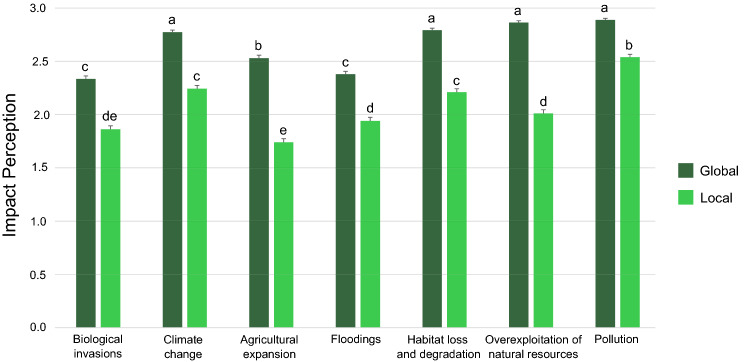
Impact perception of different threats to the environment at local and global scale. Different letters mean a significant difference (Friedman test, *P* < 0.05).

Most of the respondents considered that all threats caused medium to high impact to the environment but there was a definite inclination to think that environmental threats have a higher impact globally than locally (Q11–Q12 Supplementary Appendix S1, Fig. 2, Friedman test adjusted for ties, Critical value = 2098.541, gl = 7, *P*(Chisq) = 0). On a global scale, climate change, habitat loss and degradation, overexploitation of natural resources and pollution were ranked as the highest impact, followed by expansion of agriculture, floodings and biological invasions. On a local scale, pollution was perceived as having the highest impact on the environment, followed by climate change and habitat loss and degradation. Overexploitation of natural resources, floodings, biological invasions and expansion of agriculture ranked with lower impact (Fig. 2, Supplementary Appendix S2, Supplementary Table S2).

**Figure 3:**
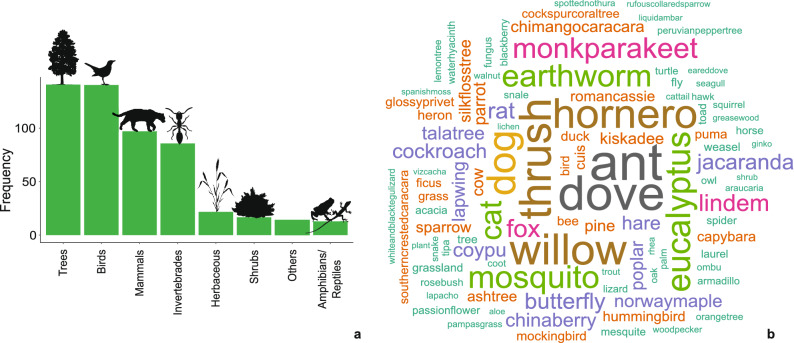
Respondent perception of biotic components of the environment, (**a**) frequency of biotic components listed by respondents grouped in eight categories, (**b**) word cloud of listed biotic components by respondents. Bigger letter size implies more frequency of presence in replies.

When respondents were asked to mention biotic components of their environment (Q13, Supplementary Appendix S1), most of them mentioned trees and birds (frequency ~ 150), followed by mammals and invertebrates (frequency ~ 100), and with low frequency (less than 20) herbaceous plants, shrubs, amphibian/reptiles and others (Fig. 3a). Considering biotic components at species level, respondents indicated 78 different species (mentioned at least five times) which were graphed in a word cloud (Fig. 3b). “Ant” and “dove” were the most frequently mentioned components followed by “willow” and the birds “hornero” and “thrush”. Interestingly, only one respondent (0.2%) mentioned humans as a biotic component of the environment. However, in the following question (Q14, Supplementary Appendix S1) when they were specifically asked if they considered humans as part of the environment, 94% responded “yes”, 2% “no” and 4% responded “maybe”. When asked to explain their reasoning behind their answer, the most common justification for these last answers (Q15, Supplementary Appendix S1) contemplated humans as part of the environment and interacting with nature and species, particularly modifying them (Supplementary Appendix S2, Supplementary Fig. S2).

### Recognition of native species and IAS

**Figure 4:**
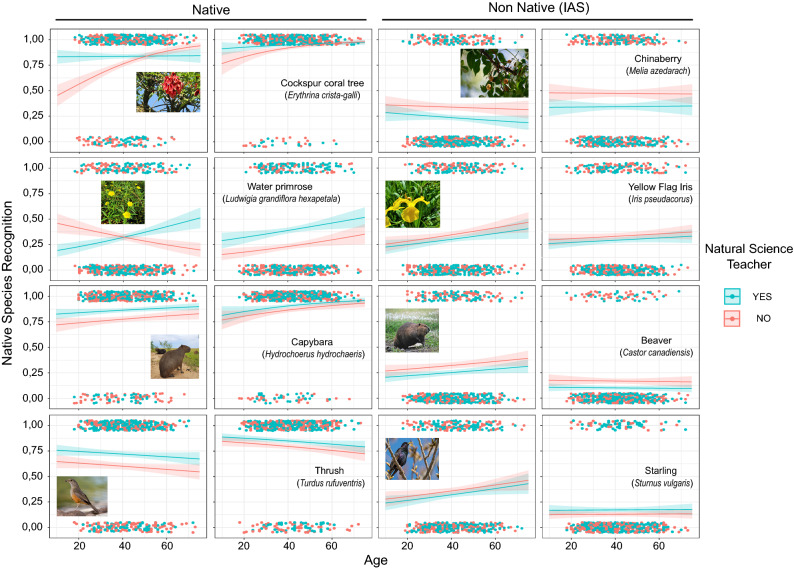
Predicted models of native and non-native species (invasive alien species, IAS) recognition from their photos and written names, considering age as an explanatory continuous variable in X axis and Natural Science Teachers (NST) of the respondents as categorical explanatory variable in two colours. Native species are shown on the left of the figure and non-native species on the right. Each frame consists of the respondent's recognition of native species offered graphed as dots (1 = native and 0 = non-native). Curves and confidence bands (95%) came from generalised linear models.

The recognition of native species based on their photographs (Q16) or species and common written name (Q17) was analysed through 112 models (Fig. 4, Table 2, Supplementary Appendix S2, Supplementary Table S3). In general, out of the seven models tested for each species in both formats (photograph and written names), the two that combined NST and Age as explanatory variables with and without interaction were selected. However, for visual comparison purposes and to contemplate one of the original aims of this research, models that consider these two variables were also graphed (Fig. 4, Table 2) for those cases where they were not the best explanatory models (Supplementary Appendix S2, Supplementary Table S3).

**Table 2:** Generalised linear model from recognition of native species. Respondents selected native/s species of photos or written names of eight species (four native species-four invasive alien species). Selection of native species (yes or no questions) was modelled considering age and if teachers were or not, Natural Science Teachers (NST). Significant differences are written in bold.

	Photography			Written name		
	Estimate	St. error	*P*	Estimate	St. error	*P*
**Cockspur coral tree (*Erythrina crista-galli*)**						
Intercept	0.344	0.551	0.244	1.090	0.653	0.095
Age	**0.045**	**0.014**	**0.001**	**0.033**	**0.016**	**0.043**
NST_yes	**2.229**	**0.833**	**0.007**	0.532	0.355	0.134
Age:NST_yes	**− 0.044**	**0.020**	**0.030**	NA	NA	NA
**Chinaberry tree (*Melia azedarach*)**						
Intercept	− 0.445	0.375	0.252	− 1.1e−01	3.5e−01	0.747
Age	− 0.006	0.009	0.513	5.4e−05	7.9e−03	0.944
NST_yes	− **0.512**	**0.194**	**0.008**	− **5.4e−01**	**1.8e−01**	**0.002**
Age:NST_yes	NA	NA	NA	NA	NA	NA
**Water primrose (*Ludwigia grandiflora* subsp. *hexapetala*)**						
Intercept	0.023	0.493	0.963	− **1.834**	**0.386**	**2.0e−06**
Age	− 0.019	0.012	0.106	0.016	0.008	0.058
NST_yes	− **1.686**	**0.705**	**0.017**	**0.746**	**0.195**	**1.7e−04**
Age:NST_yes	**0.041**	**0.017**	**0.012**	NA	NA	NA
**Yellow flag iris (*Iris pseudacorus*)**						
Intercept	− **1.309**	**0.353**	**2.0e−04**	− **0.914**	**0.368**	**0.013**
Age	0.014	0.008	0.087	0.005	0.008	0.515
NST_yes	NA	NA	NA	− 0.178	0.188	0.342
Age:NST_yes	NA	NA	NA	NA	NA	NA
**Capybara (*Hydrochoerus hydrochaeris*)**						
Intercept	0.848	0.437	0.052	0.906	0.520	0.081
Age	0.010	0.010	0.336	0.024	0.013	0.056
NST_yes	**0.609**	**0.231**	**0.009**	0.394	0.276	0.154
Age:NST_yes	NA	NA	NA	NA	NA	NA
**Beaver (*Castor canadiensis*)**						
Intercept	− **1.113**	**0.378**	**0.003**	− **1.518**	**0.491**	**0.002**
Age	0.009	0.084	0.300	− 0.002	0.011	0.868
NST_yes	− 0.344	0.194	0.076	− **0.584**	**0.259**	**0.024**
Age:NST_yes	NA	NA	NA	NA	NA	NA
**Thrush (*Turdus rufuventris*)**						
Intercept	0.671	0.360	0.062	**1.821**	**0.446**	**4.5e−05**
Age	− 0.007	0.008	0.421	− 0.011	0.010	0.247
NST_yes	**0.536**	**0.185**	**0.004**	0.370	0.228	0.105
Age:NST_yes	NA	NA	NA	NA	NA	NA
**Starling (*Sturnus vulgaris*)**						
Intercept	− **1.095**	**0.361**	**0.002**	− **1.932**	**0.480**	**5.6e−05**
Age	0.013	0.008	0.110	0.001	0.011	0.931
NST_yes	− 0.166	0.184	0.367	0.329	0.247	0.183
Age:NST_yes	NA	NA	NA	NA	NA	NA

In general, recognitions were more accurate when the respondents saw written names rather than photos, both for native species and IAS (Fig. 4). Most respondents recognised all native species, cockspur coral tree, capybara and thrush as such, except for water primrose (Supplementary Appendix S2, Supplementary Table S1). Different results were obtained when presented with the photo or the written name of the cockspur coral tree (*E. crista-galli*, Fig. 4). NST recognition of the photo remained almost constant close to one (~ 0.8), whereas non-NST increased with age (significant interaction *P* < 0.5, Table 2, Supplementary Table S3). When presented with the species' written name recognition only increased significantly with age (no interaction model, Fig. 4, Table 2, Supplementary Appendix S2, Supplementary Table S3). Out of the four native species offered, water primrose (*L. g.* subsp. *hexapetala*) was the least selected as such. The recognition of this aquatic plant differed between the photo and the species’ written name. The best models that explained water primrose’s photo recognition as native were the one with NGEO as explanatory variable and followed by the one with NST and Age (model with interaction, Fig. 4, Table 2, Supplementary Appendix S2, Supplementary Table S3). NST recognised water primrose as native increasingly with age whereas for non-NST recognition decreased. For the species' written name NST recognised water primrose as native more than non-NST (no interaction model, Fig. 4, Table 2, Supplementary Appendix S2, Supplementary Table S3). For capybaras’ photo and written name (*H*. *hydrochaeris*), NST recognised its native condition better than non-NST. Also, the species’ written name had a marginal recognition increase with age (no interaction models, Fig. 4, Table 2, Supplementary Appendix S2. Supplementary Table S3). In the case of the thrush photo (*T. rufuventris*), NST recognised the species as native better than non-NST. However, for the written name no significant differences between NST and non-NST were observed (no interaction models, Fig. 4, Table 2, Supplementary Appendix S2, Supplementary Table S3).

On the other hand, IAS were least chosen as native (Native Species Recognition Value < 0.5, Fig. 4). Accurately, NST did not select the chinaberry tree (*M. azedarach*) as native compared to the non-NST regardless of respondents' age, both for the photo and written name (no interaction model, Fig. 4, Table 2, Supplementary Appendix S2, Supplementary Table S3). Recognition of the yellow flag iris (*I. pseudacorus*) photo as native was low but increased with respondent’s age regardless of their NST condition (no interaction model, Fig. 4, Table 2, Supplementary Appendix S2, Supplementary Table S3). For this species' written name, the null model had the lowest AIC, implying that recognition was free running, followed by the model with NST condition even though no parameters were significant (Fig. 4, Table 2, Supplementary Appendix S2, Supplementary Table S3). Respondents did not choose the beaver photo (*C. canadiensis*) as a native species regardless of their NST condition and age. On the other hand, with the written species name, NST did not select the beaver as native compared to the non-NST (no interaction models, Fig. 4, Table 2, Supplementary Appendix S2, Supplementary Table S3). Finally, regarding the IAS starling (*S. vulgaris*), both photo and the written name had low values of native species recognition and showed no significant differences between NST and non-NST nor Age (no interaction models, Fig. 4, Table 2, Supplementary Appendix S2, Supplementary Table S3).

### Biological invasions awareness

**Figure 5:**
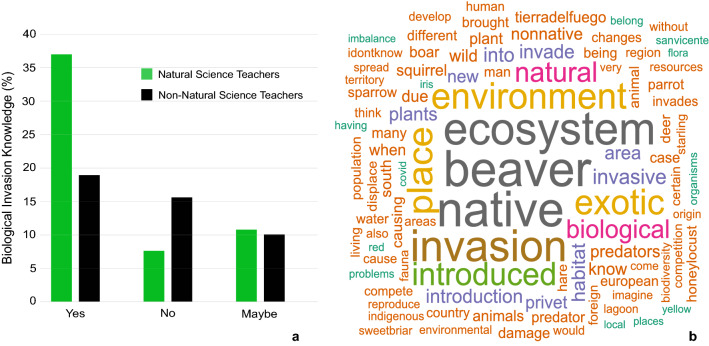
Biological invasions awareness from education community, (**a**) perception knowledge of Natural Science Teacher (NST) and non-NST, and (**b**) word cloud from text mining analysis of respondent knowledge about biological invasion. Bigger letter size implies more frequency of presence in replies.

Respondents showed great awareness of biological invasions (Q18, Supplementary Appendix S1) and it was more noticeable on NST (Chi-squared = 38.812, df = 2, *P* (Chisq) = 3.733e−09, Fig. 5a, Supplementary Appendix S2, Supplementary Fig. S3). From a total of 532 respondents, 298 (56%) knew what biological invasions are, 111 (21%) answered “Maybe” and 123 (23%) had never heard about them. From those who knew, two out of three (66%) were NST (Fig. 5a). Such a ratio was reverted on those who did not know the concept, 2/3 were non-NST (66%, Fig. 5a). Finally, those who were not sure if they knew the concept were balanced between NST and non-NST (Fig. 5a).

People expressed with more detail what they knew about biological invasions and examples in Q19 (Supplementary Appendix S1, Fig. 5b). The most frequently mentioned words were “beaver”, “native”, “ecosystem”, “exotic”, “invasion”, “environment”, “introduced” and “natural”. The word “beaver” was the IAS most frequently mentioned for both, NST and non-NST (Fig. 5b, Supplementary Appendix S2, Supplementary Figs. S4–S5). Other IAS like “sparrow” (*Passer domesticus*), “deer” (*Cervus elaphus*), “privet” (*Ligustrum* spp.), “squirrel” (*Callosciurus erythraeus*), “hare” (*Lepus europaeus*), “boar” (*Sus scrofa*), “parrot” (*Myiopsitta monachus*), and “honey locust” (*Gleditsia triacanthos*) were more frequently mentioned in NST answers (Supplementary Appendix S2, Supplementary Fig. S4).

## Discussion

To our knowledge, this is the first study on understanding and perception of environmental issues of a teacher community focusing on native species, IAS and the awareness of biological invasions. A key aspect we found in this study is the difference between NST and non-NST regarding environmental issues.

The surveyed educational community perceived the impact of different threats to the environment to be greater globally than locally, highlighting pollution, habitat loss and degradation and overexploitation of natural resources. These drivers were also considered the most detrimental in another study with student teachers, alluding responsibility to demographic growth of the human population^23^. Biological invasions, one of the main drivers of biodiversity loss^5^, was considered less important both globally and locally in the present study as well as in other studies^9,23^. This could be a consequence of the absence, in the school curricula, of IAS threats to the environment^4,7,10^.

People trained in science and environmental areas have a better knowledge and perception of native species and IAS^7,24^. Here, NST recognised native species as such better than non-NST, and did not select correctly non-native species or IAS. These results were similar to other studies on perception of IAS^10,16,25,26^, and to others on people’s awareness of natural disasters^27^. Similarly, Remmele and Lindemann-Matthies^10^ found that biology teacher students identified IAS better than teacher students of other subjects, and more often selected management strategies to control them.

In our study, the educational community performed better in recognising native species when they chose between written names rather than photographs of the same species. Both NST and non-NST recognised the cockspur coral tree or *ceibo* as native with greater precision when they saw the written name, probably in accordance with its cultural value and oral and written spread. This species is important as it is the national flower, widely represented in literature and bill illustrations^28^. We had previously conducted outreach activities with similar results (Supplementary Appendix S3), where cockspur coral tree was also better recognised when teachers were offered the written name rather than the photograph. Nevertheless, recognising the name does not necessarily mean recognising the tree. On the other hand, IAS such as starling and beaver were considered as not native by most of the respondents. In particular, beavers are one of the main IAS vertebrates in Argentina. They are very well known for building dykes in important water courses of Tierra del Fuego Province (south of Argentina) with their environmental damaging consequences downstream^29^. This dilemma seems to lay on a variety of explanations, depending on each case, from how images or words (names in this case) are processed in the brain to the absence of a real search image to recognise and compare. This could also be attributed to how nature is taught, understood or perceived, from theoretical aspects in a classroom^3^. In Argentina, outdoor learning or hands-on practicing is not necessarily a school requirement and therefore it could be expected that people do not recognise photographs of nature, animals and plants. Traditional teaching methods often isolate classroom environments from relevant local scientific topics, separating students’ learning from actual processes and patterns that occur in nature^30^.

We found that the age of the respondents influenced the recognition of some species. However, there is not a clear pattern on how age influences not only NST and non-NST but also how it marks a difference between IAS and native species. A similar age-related result was found in people’s perceptions of alien parakeets^24,31^. In concordance, Fitzgerald^32^ also found that older people are reluctant to choose an IAS while we found the opposite pattern with the invasive yellow flag iris. This may be showing some changes that occurred in education and cultural aspects in the last decades regarding teaching methodologies or any other approaches to science and environmental themes. These inconclusive results should encourage more research in this aspect.

Biological invasions are becoming widely recognised and the awareness of their impacts is increasing^7,20^. More than half of the respondents were aware of biological invasions, especially NST who recognised more plant and animal IAS (e.g., privet, beaver, deer, squirrel, boar, parrot, starling) compared to the non-NST group. Most of these IAS are included in the Project of IAS National Strategy (ENEEI, acronym in Spanish), coordinated by the National Ministry of Environment and Sustainable Development, which recorded similar percentages of interest, awareness and recognition of IAS^20^. Interesting results could be obtained by conducting similar studies between regions with shared IAS problems (e.g., *I. pseudacorus* and *M. azedarach* invade Argentina and South Africa)^33^ and regions that share native and invasive species in a reciprocal way (e.g., *L. g.* subsp. *hexapetala* native to South America and invasive in Europe; and *I. pseudacorus* native to Europe and invasive in South America)^34,35^. Biological invasions will continue to increase—often at accelerating rates—in the next three decades^36^ impacting on biodiversity, ecology, and socio-economic and human health systems^22^ and demanding urgent management policies^20,37–39^. It is relevant because acceptance of IAS management (e.g., control or removal techniques) could be related to having a background of environmental knowledge^4,10,26^. Results from these studies could help to obtain greater support and compromise from a multiple-stakeholder approach: policy makers, scientists, teachers, students, non-formal educators and the general public^2,3,10^.

From our results, we encourage improvements in environmental education for currently working and future teachers. Knowledge and recognition of native species, and IAS and their potential threats should be included in formal and non-formal educational curricula using friendly novel tools to capture also the attention of the new generations. Citizen Science projects, defined as researchers engaging the public in data collection to increase scientific knowledge^40^, related to IAS are on the rise^36,41–44^. They can improve communication with the public so that it can bring changes in participants’ knowledge, skills, attitudes, and behaviours^45–48^. For students it can be an opportunity to participate in authentic scientific researches, thereby increasing students’ interest, motivation and attitudes towards science or the environment^49^.

In the context of formal education, curricula should promptly acquire changes to reach the expected goals successfully, including hands-on learning activities and formal outside learning, not only in subjects like biology, but also in a cross-curricular approach at schools and universities^3,4,7,10,22^. For instance, a stronger focus on species taxonomy might help teachers to share this knowledge in class^10,37,38^. Activities like discussion groups, role play and formal outdoor learning, proved to be useful to generate greater involvement in various scientific topics^2,7,51^, and prevent gender gaps in sciences^52^. As reported by Verbrugge et al.^3^ and Sosa et al.^53^ students and teachers can act as multiplying agents who can contribute to solving environmental problems. IAS need to be treated in a multiple and contextualised way since environmental problems involve and are related to numerous aspects of society^50^.

Non-formal education is related to the cultural identity of societies and their environment. Amplifying NGEO campaigns, botanic gardens, museums, wildlife parks contribution, park rangers’ tasks, and programs of reintroductions of locally-native-extinct species^54^ (e.g., anteaters, jaguar) are fundamental tools to increase and spread the environmental knowledge and awareness to the whole community^2,55,56^.

## Conclusions

In accordance with the UNDP objectives, all educators (trained or not in environmental issues) should acquire the necessary knowledge and skills to promote sustainable development. Campaigns to increase awareness on environmental issues and perception of native flora and fauna of the community should be improved. We need to foster capacity-building, encourage two-way communication between educators and scientists to impact public participation right through from kindergarten to the university levels. We must strengthen environmental awareness-raising of people in relation to the role of humans as a part of nature, to find solutions for environmental threats, such as biological invasions. To achieve this, cross-curricular work, including co-development of educational material, is necessary in environmental issues to empower educators, both formal and informal, in the whole of society.

## Materials and methods

### Survey planning and data collection

Argentina is a country extended along 18 ecoregions^57^ with high biodiversity, and in the last decade, the number of IAS has increased (> 1000 spp.) causing an estimated loss of US$ 3.4 billion, equivalent to 0.63% of the gross domestic product^58^. We investigated the knowledge and perception of native species and IAS of the educational community in Argentina (~ 1,400,000 teachers)^59^. We designed a survey to query a sample of individuals from this educational community regarding their perception of the environment, recognition of native species and IAS, and their understanding of biological invasions. It consisted of an online semi-structured questionnaire of 19 sequenced questions using Google Forms with an introductory paragraph indicating the purpose of the study. The questionnaire covered four main areas: (1) characterisation of the respondents, (2) perception of the environment (biotic components and environmental threats), (3) recognition of native species and IAS, and (4) awareness of biological invasions (Supplementary Appendix S1). We used a “snowball” sampling approach through emails, social media platforms (Twitter, Facebook, Instagram and Whatsapp), and web sites from Universidad Nacional de Hurlingham (UNAHUR, unahur.edu.ar) and FuEDEI (fuedei.org). Responses were recorded during 110 days (June 5–September 23, 2020) under COVID-19 pandemic lockdown. All methods were carried out in accordance with relevant guidelines and regulations. This study was approved by UNAHUR and FuEDEI, and formal written consent was not required according to guidelines and regulations, therefore the UNAHUR Bioethics Committee confirmed the non-need for informed consent in the case of an anonymous survey like this work.

### Characterisation of the respondent

To characterise the socio-demographic profile, the respondents were asked to answer: their age, city/town, education level and how they are related to education. If they were teachers we asked: subject, level and for how long they have been teaching (Supplementary Appendix S1). Later in the analysis, respondents were divided into two groups: NST (Biology, Mathematics, Physics and Chemistry) and non-NST (e.g., Art, Social Science, Literature, Physical Education).

### Perception of the environment

In this section, we asked respondents to rate from 1 to 3 (1 being low and 3 high) the impact certain threats (e.g., climate change, pollution, flooding, habitat and loss degradation among others) have on the environment at local (their community) and global scale (Q11–Q12, Supplementary Appendix S1). Ranks of threats were analysed by Friedman test in *agricolae* package^60^ (version 1.3-3), followed by multiple comparison through LSD test.

We asked them to list three biotic components of the environment (Q13, Supplementary Appendix S1). We then asked if they considered human beings as a part of the environment and why (Q14–Q15, Supplementary Appendix S1). We grouped biotic components into eight taxonomic categories: trees, birds, mammals, invertebrates, herbaceous, shrubs, amphibians and reptiles, and others; and visualised their frequency through histograms. Biotic components listed (Q13) and answers as to why humans are part of the environment (Q15) were respectively analysed through text mining analysis with *tm* package^61^ (version 0.7-7) in R software^62^ and finally graphed the 100 most frequent words in world clouds.

### Recognition of native species and IAS

We estimated the respondents’ recognition of native species through Q16 and Q17 (Supplementary Appendix S1). Respondents were asked to recognise native species from the Argentine environment by two different approaches: eight photographs (Q16) and eight written names (common and scientific) of the same species (Q17). Each question had four native and four non-native species randomly distributed (Google Forms tool).

The native species were: cockspur coral tree (*Erythrina crista-galli, ceibo* in Spanish), water primrose (*Ludwigia grandiflora* subsp. *hexapetala, duraznillo de agua* in Spanish), capybara (*Hydrochoerus hydrochaeris, carpincho* in Spanish), and thrush (*Turdus rufuventris, zorzal* in Spanish). We choose the cockspur coral tree because it is the Argentine national flower^63^ and water primrose as it is a native aquatic plant from South America not commonly known^64^. The capybara was chosen because it is the biggest rodent in the world and a typical species of the largest wetland in Argentina (Del Plata Basin)^65^, and the thrush is one of the most abundant birds in urban and non-urban areas of the country^66^.

The non-native species (in this case also IAS) selected belong to the same taxonomic or environmental group as the natives (trees, aquatic plants, mammals and birds). They were chinaberry tree (*Melia azedarach, paraíso* in Spanish), yellow flag iris (*Iris pseudacorus, lirio amarillo* in Spanish), beaver (*Castor canadiensis, castor* in Spanish), and starling (*Sturnus vulgaris, estornino* in Spanish).

The selected IAS were recently listed as nationally “Restricted and mandatory control species”^39^. The chinaberry tree is one of the most allergenic trees in Argentina that was highly used as an ornamental tree in public spaces^67^. The beaver is one of the most harmful mammals in southern Argentina^29^. The starling was selected for its great capacity to adapt and reproduce in urban and peri-urban areas, forming flocks of thousands of individuals^68^. Finally, yellow flag iris is one of the most recent and fast-expanding IAS in most Argentine wetlands, appreciated as an ornamental for its yellow flowers^69^.

We modelled the recognition of native species using generalised linear models (GLM) based on binomial distribution with a *log link* function, considering recognition of native species (photographs or written names) as the response variable with and without interaction of the explanatory variables (Age, NST, Education level, and NGEO membership) (Supplementary Appendix S2, Supplementary Table S3). Model selection was based according to the lowest AIC (minimum model) and Delta AIC (< 2), parsimony principle, and considering the objectives of this studies (Supplementary Appendix S2, Supplementary Table S3).

### Biological invasions awareness

Perception of biological invasions was addressed in Q18 and Q19 (Supplementary Appendix S1). We asked if respondents were aware of the concept of biological invasions, then analysed and compared the frequency of yes–no-maybe answers using Pearson’s Chi-squared test. In Q19 we requested more details and examples. Comments were analysed through text mining analysis with *tm*^61^ package in R software^62^ and finally the 100 most frequent words graphed in a world cloud.

## Supplementary Information

Supplementary Information.
