# Supplementary material for: The educational community and its knowledge and perceptions of native and invasive alien species
Source: Sci Rep. 2021 Nov 2;11:21474. doi: 10.1038/s41598-021-00683-y (PMC8563966; doi:10.1038/s41598-021-00683-y)
Supplement: Supplementary file 1 — Supplementary Information. [file 41598_2021_683_MOESM1_ESM.pdf]

SUPPLEMENTARY INFORMATION

The educational community and its knowledge and perceptions of native and invasive alien species

Alejandro J. Sosa[1,2], Nadia L. Jiménez[1,2], Ana C. Faltlhauser[1,2], Tomás Righetti[1,2], Fernando McKay[1], Octavio A. Bruzzone[3], Iris Stiers[4], Adriana Fernández Souto[5]

1 *Fundación para el Estudio de Especies Invasivas (FuEDEI). Simón Bolívar 1559, (B1686EFA), Hurlingham, Buenos Aires, Argentina.*

2 *Consejo Nacional de Investigaciones Científicas y Técnicas (CONICET). Godoy Cruz 2290, (C1425FQB), CABA, Argentina.*

3 *Agroecology, Environment and Systems Group, Instituto de Investigaciones Forestales y Agropecuarias de Bariloche (IFAB), INTA-CONICET. Modesta Victoria 4450, CC 277, (R8400XAC), San Carlos de Bariloche, Río Negro, Argentina.*

4 *Multidisciplinary Institute for Teacher Education (Science & Technology, MILO), Vrije Universiteit Brussel. Pleinlaan 9, (1050), Brussels, Belgium.*

5 *Instituto de Educación, Universidad Nacional de Hurlingham (UNAHUR). Teniente Origone 151, (B1688AAA), Hurlingham, Buenos Aires, Argentina.*

Appendix S1. Questionnaire

Appendix S2. Responses to the questionnaire

Appendix S3. Preliminary and complementary studies on species recognition

Appendix S1. Questionnaire (Translated from Spanish)

I. Characterisation of the respondent

1. Age: ______

2. Where do you live? (Province, Department/State, City) ______

3. Are you related to the teaching field? □ Yes, I am a teacher □ Yes, I am a student of a teaching career □ Yes, I am a retired teacher □ No □ Others ______

4. Which education level do you have? (Qualifications) □ Incomplete Secondary School □ Complete Secondary School □ Incomplete University □ University graduate □ Incomplete Postgraduate University □ Complete Postgraduate University

5. How long have you been teaching? ______

6. Indicate where do/did you teach? (You can choose more than one) □ Kindergarten □ Primary School □ Secondary School □ University □ Special Education □ Others ______

7. In which area/s did/do/will you teach? (You can choose one or more options) □ Mathematics □ Literature □ Natural Science □ Social Science □ Art □ Physical Education □ Foreign language □ Others ______

8. Are you a member or do you participate in any NGEO (non-governmental environmental organizations)? □ Yes □ No □ Others ______

9. Have you ever visited a protected area, such as a municipal/provincial/national reserve or park? How often did you or do visit it? □ Yes, weekly □ Yes, monthly □ Yes, several times a year □ Yes, at least once a year □ Yes, every 3-5 years □ Never □ Others ______

10. Do you do or do you like to do sports or outdoor recreational activities? (You can choose more than one option) □ Fishing □ Hunting □ Nautical sports □ Gardening/Horticulture □ Wildlife watching (i. e., bird-watching) □ Outdoor walks □ I do not do outdoor activities □ Others ______

II Perception of the Environment

11. We know that our environment is threatened by different factors. Could you tell us what impact you think the following issues have on the environment WORLDWIDE/GLOBAL scale (Being 1 low impact, 2 medium impact and 3 high impact).

Issues	1	2	3
Flooding			
Climate change			
Biological invasions			
Pollution			
Habitat loss and degradation			
Agricultural expansion			
Overexploitation of natural resources			

12. And in our surroundings? Could you tell us what impact you think the following issues have on your LOCAL environment where your school or community is? (Being 1 low impact, 2 medium impact and 3 high impact).

Issues	1	2	3
Flooding			
Climate change			
Biological invasions			
Pollution			
Habitat loss and degradation			
Agricultural expansion			
Overexploitation of natural resources			

13. In ecosystems we find different biotic components (living beings such as animals, plants, fungi, microorganisms). Could you name THREE biotic components from the environment where you live? Try to write their names. Example: Willow instead of tree, Red fire ant instead of insect. Other examples: hornero, thrush, cat, earthworm, linden, rose).
Biotic component 1. ______, Biotic component 2. ______, Biotic component 3. ______

14. Do you consider that the humans are part of the ecosystem where you live? □ Yes □ No □ Maybe

15. Could you explain the reason why you selected the last answer? ______

III: Recognition of Native and Invasive Alien Species

16. Let's play. The following photographs correspond to a group of native and exotic species from the environments of our country (Argentina). Could you indicate which you think are NATIVE SPECIES? (Photographs appear at random in each questionnaire).

□ 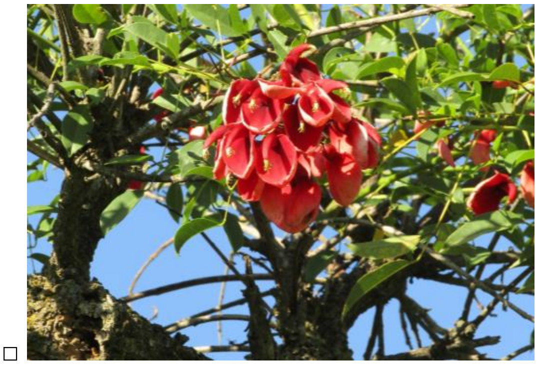
Photo by Ana Faltlhauser

□ 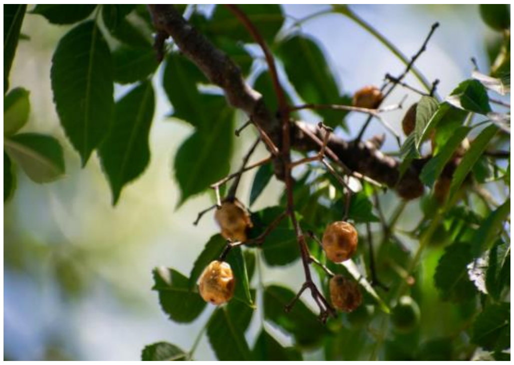
Photo by Nicolás Vernazza

□ 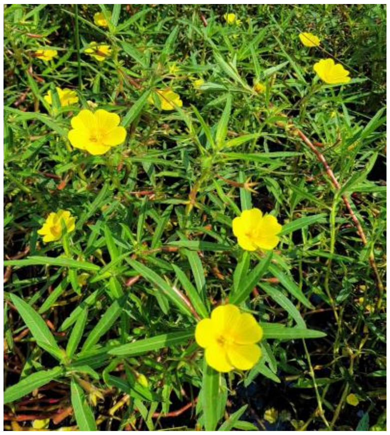
Photo by Ana Faltlhauser

□ 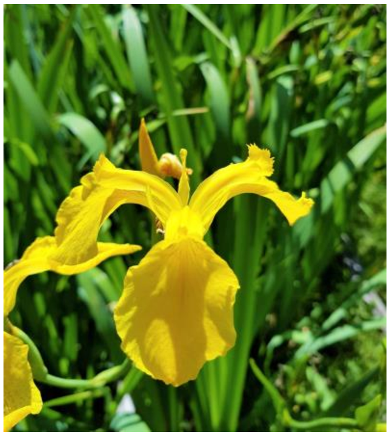
Photo by Ana Faltlhauser

□ 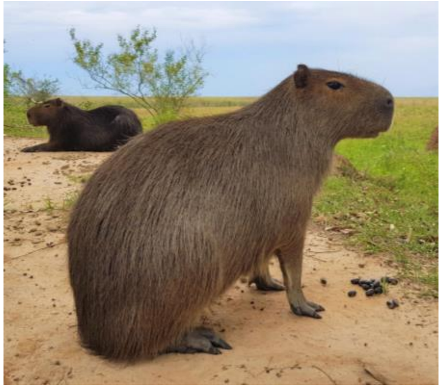
Photo by Ana Faltlhauser

□ 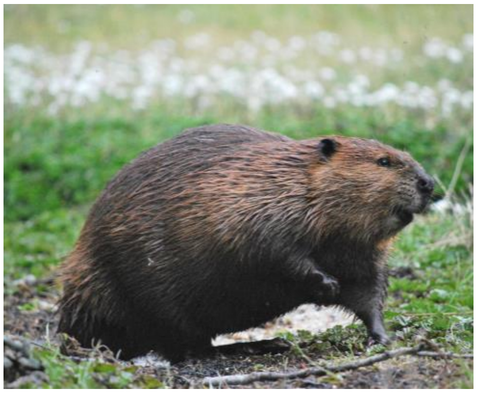
Photo by “Moncho” Alvarado

□ 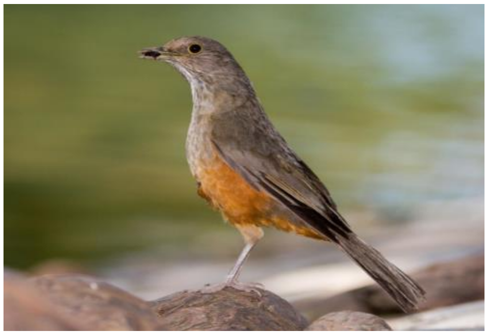
Photo by Adrian Grilli

□ 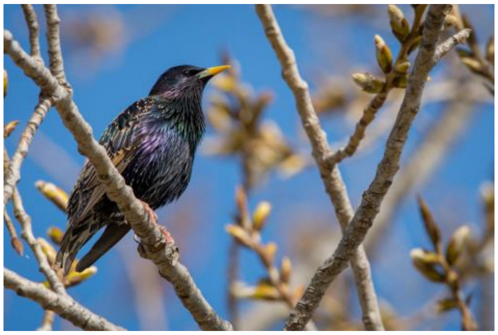
Photo by Adrian Grilli

17. The following are common and scientific names of native and exotic species found in our country. Which are NATIVE SPECIES? (Written names appear at random in each questionnaire).

□ Cockspur coral tree (*Erythrina crista-galli*) □ Chinaberry tree (*Melia azedarach*) □ Water primrose (*Ludwigia grandiflora* subsp. *hexapetala*) □ Yellow Flag Iris (*Iris pseudacorus*) □ Capybara (*Hydrochoerus hydrochaeris*) □ Beaver (*Castor canadiensis*) □ Thrush (*Turdus rufuventris*) □ Starling (*Sturnus vulgaris*)

IV. Biological Invasions Awareness

18. Have you ever heard about biological invasions? Do you know what they mean? □ Yes □ No □ Maybe

19. We would like you to tell us a little more about what you know about BIOLOGICAL INVASIONS or if you know any examples ______

Appendix S2. Responses to the questionnaire

Sampling methodology, estimating sample size

The target population, teachers of Argentina, is estimated in around 1400000 according to Ministry of Education of Argentina[1]. To estimate minimal sample (S) of our survey we used the following equation, modified from D'Ancona[2]:

Equation S1.

$$S = \frac{\frac{Z^2 P(1-P)}{e^2}}{1 + \left(\frac{Z^2 P(1-P)}{e^2 N}\right)}$$

Where N=population size, e=error, z=score of standard deviation.

For this purpose, N=1,400,000, e=0.05, z=1.96. So, we expected to have at least 385 responses with 95% of confidence level and 5% of margin error.

References

1. Ministerio de Educación de la República Argentina. Base de Datos-Información Estadística. *Base de Datos por Escuela 2019* https://www.argentina.gob.ar/educacion/evaluacion-informacion-educativa/bdd (2019).
2. D'Ancona, M. Á. *Metodología cuantitativa: estrategias y técnicas de investigación social*. (Editorial Síntesis SA, 1996).

Table S1. Descriptive statistics of the responses of the questionnaire.

Variables	Min.	1st Quartile	Median	Mean	3rd Quartile	Max
Age	18.00	33.00	40.00	41.21	49.25	73.00
Years of teaching	0.00	5.00	10.00	12.89	20.00	54.00
Educational level	1.00	4.00	4.00	4.10	4.00	6.00
Natural Science Teacher	0.00	0.00	1.000	0.55	1.00	1.00
NGEO member	0.00	0.00	0.00	0.15	0.00	1.00
Protected areas frequency	0.00	2.00	3.00	2.52	3.00	3.00
Flooding W	1.00	2.00	2.00	2.38	3.00	3.00
Climate change W	1.00	3.00	3.00	2.77	3.00	3.00
Biological Invasions W	1.00	2.00	2.00	2.33	3.00	3.00
Pollution W	1.00	3.00	3.00	2.89	3.00	3.00
Habitat destruction W	1.00	3.00	3.00	2.79	3.00	3.00
Agriculture W	1.00	2.00	3.00	2.54	3.00	3.00
Overexploitation NR W	1.00	3.00	3.00	2.86	3.00	3.00
Flooding L	1.00	1.00	2.00	1.95	3.00	3.00
Climate change L	1.00	2.00	2.00	2.25	3.00	3.00
Biological Invasions L	1.00	1.00	2.00	1.87	2.00	3.00
Pollution L	1.00	2.00	3.00	2.55	3.00	3.00
Habitat destruction L	1.00	2.00	2.00	2.21	3.00	3.00
Agriculture L	1.00	1.00	2.00	1.74	2.00	3.00
Overexploitation NR L	1.00	1.00	2.00	2.00	3.00	3.00
Capybara P	0.00	1.00	1.00	0.83	1.00	1.00
Capybara W	0.00	1.00	1.00	0.89	1.00	1.00
Beaver P	0.00	0.00	0.00	0.28	1.00	1.00
Beaver W	0.00	0.00	0.00	0.13	0.00	1.00
Cockspur coral tree P	0.00	1.00	1.00	0.80	1.00	1.00
Cockspur coral tree W	0.00	1.00	1.00	0.93	1.00	1.00
Water primrose P	0.00	0.00	0.00	0.32	1.00	1.00
Water primrose W	0.00	0.00	0.00	0.32	1.00	1.00
Starling P	0.00	0.00	0.00	0.34	1.00	1.00
Starling W	0.00	0.00	0.00	0.15	0.00	1.00
Yellow Flag Iris P	0.00	0.00	0.00	0.33	1.00	1.00
Yellow Flag Iris W	0.00	0.00	0.00	0.31	1.00	1.00
Chinaberry tree P	0.00	0.00	0.00	0.28	1.00	1.00
Chinaberry tree W	0.00	0.00	0.00	0.40	1.00	1.00
Thrush P	0.00	0.00	1.00	0.67	1.00	1.00
Thrush W	0.00	1.00	1.00	0.82	1.00	1.00
Invasion knowledge	0.00	1.00	1.00	0.97	1.00	2.00

W = world (global), L = local, NR = Natural Resources

Figure S1. Histogram of respondents' ages

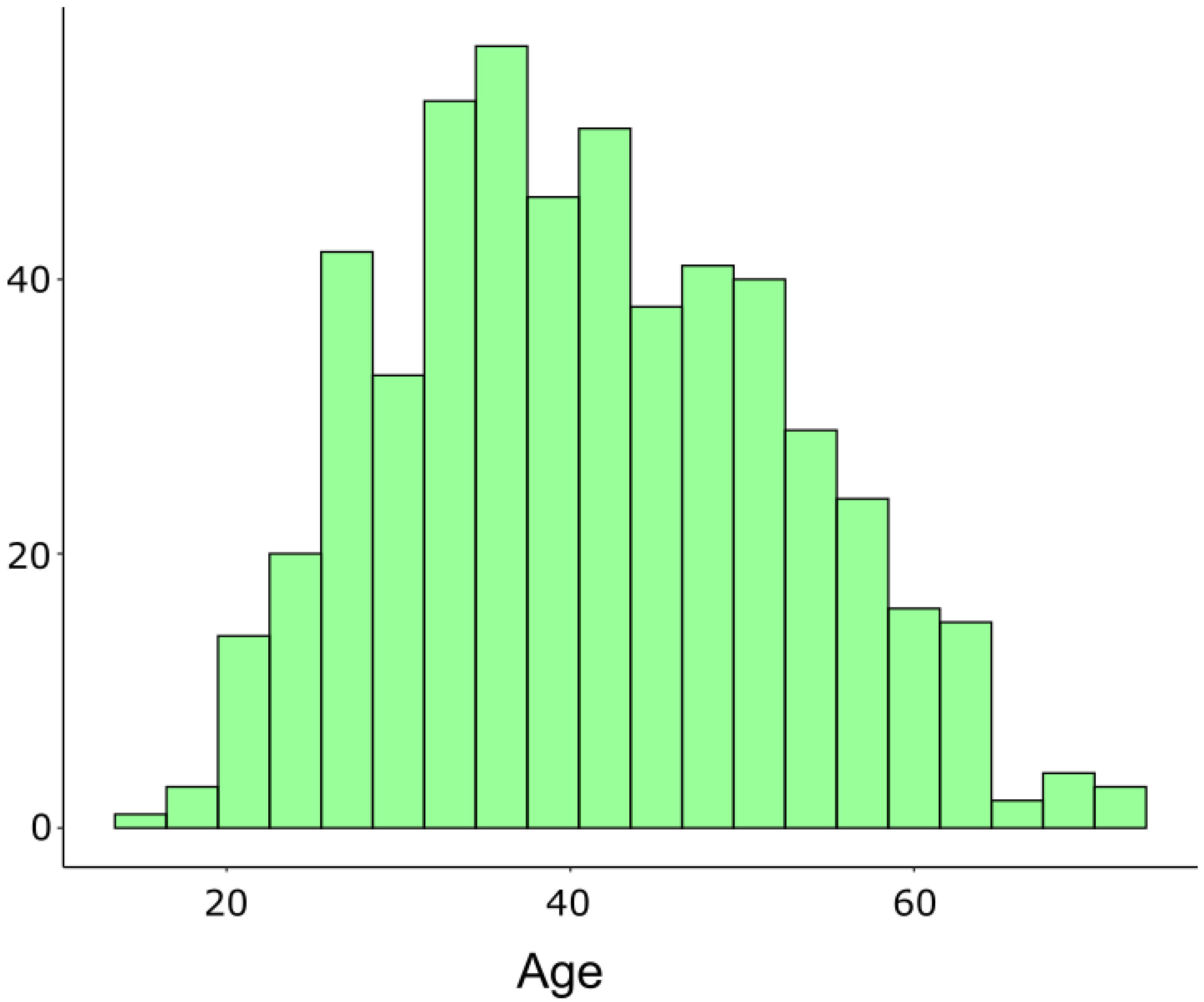

Figure S2. Most frequently appearing (or used) words (100) are utilized to explain why humans are another living component of the environment. Words are displayed in a word cloud where bigger letter size implies more frequency of presence in replies.

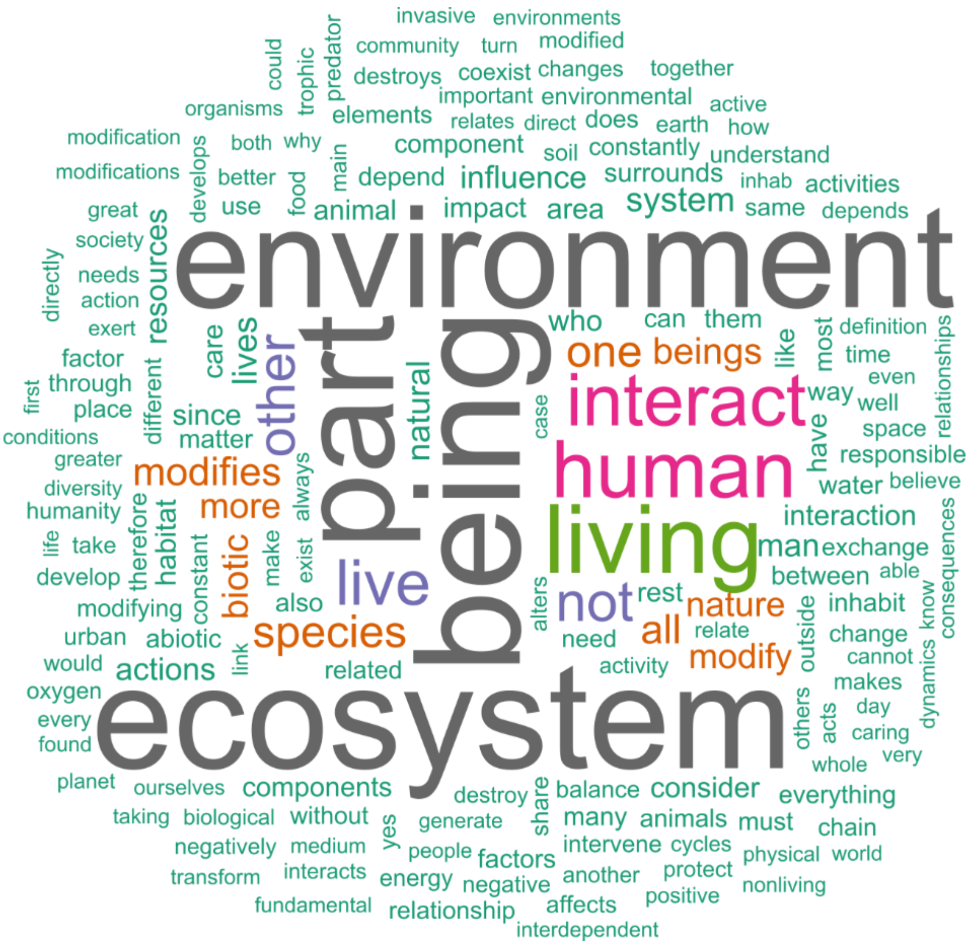

Table S2. Multiple comparisons between groups after the Friedman test. Multiple-paired comparisons and their observed differences and critical differences for a statistical significance.

Comparisons	Observed Difference	Critical Difference	Difference
Flooding_W-Climate_change_W	1171.5	471.5088	*
Flooding_W-Biol_Invasions_W	136.5	471.5088	NS
Flooding_W-Pollution_W	1526.5	471.5088	*
Flooding_W-Habitat_destruction_W	1224	471.5088	*
Flooding_W-Agriculture_W	499	471.5088	*
Flooding_W-Overexploitation_NR_W	1443.5	471.5088	*
Flooding_W-Flooding_L	1014	471.5088	*
Flooding_W-Climate_change_L	318	471.5088	NS
Flooding_W-Biol_Invasions_L	1294.5	471.5088	*
Flooding_W-Pollution_L	541	471.5088	*
Flooding_W-Habitat_destruction_L	369	471.5088	NS
Flooding_W-Agriculture_L	1475	471.5088	*
Flooding_W-Overexploitation_NR_L	881.5	471.5088	*
Climate_change_W-Biol_Invasions_W	1308	471.5088	*
Climate_change_W-Pollution_W	355	471.5088	NS
Climate_change_W-Habitat_destruction_W	52.5	471.5088	NS
Climate_change_W-Agriculture_W	672.5	471.5088	*
Climate_change_W-Overexploitation_NR_W	272	471.5088	NS
Climate_change_W-Flooding_L	2185.5	471.5088	*
Climate_change_W-Climate_change_L	1489.5	471.5088	*
Climate_change_W-Biol_Invasions_L	2466	471.5088	*
Climate_change_W-Pollution_L	630.5	471.5088	*
Climate_change_W-Habitat_destruction_L	1540.5	471.5088	*
Climate_change_W-Agriculture_L	2646.5	471.5088	*
Climate_change_W-Overexploitation_NR_L	2053	471.5088	*
Biol_Invasions_W-Pollution_W	1663	471.5088	*
Biol_Invasions_W-Habitat_destruction_W	1360.5	471.5088	*
Biol_Invasions_W-Agriculture_W	635.5	471.5088	*
Biol_Invasions_W-Overexploitation_NR_W	1580	471.5088	*
Biol_Invasions_W-Flooding_L	877.5	471.5088	*
Biol_Invasions_W-Climate_change_L	181.5	471.5088	NS
Biol_Invasions_W-Biol_Invasions_L	1158	471.5088	*
Biol_Invasions_W-Pollution_L	677.5	471.5088	*
Biol_Invasions_W-Habitat_destruction_L	232.5	471.5088	NS
Biol_Invasions_W-Agriculture_L	1338.5	471.5088	*
Biol_Invasions_W-Overexploitation_NR_L	745	471.5088	*
Pollution_W-Habitat_destruction_W	302.5	471.5088	NS
Pollution_W-Agriculture_W	1027.5	471.5088	*
Pollution_W-Overexploitation_NR_W	83	471.5088	NS
Pollution_W-Flooding_L	2540.5	471.5088	*
Pollution_W-Climate_change_L	1844.5	471.5088	*
Pollution_W-Biol_Invasions_L	2821	471.5088	*
Pollution_W-Pollution_L	985.5	471.5088	*

Pollution_W-Habitat_destruction_L	1895.5	471.5088	*
Pollution_W-Agriculture_L	3001.5	471.5088	*
Pollution_W-Overexploitation_NR_L	2408	471.5088	*
Habitat_destruction_W-Agriculture_W	725	471.5088	*
Habitat_destruction_W-Overexploitation_NR_W	219.5	471.5088	NS
Habitat_destruction_W-Flooding_L	2238	471.5088	*
Habitat_destruction_W-Climate_change_L	1542	471.5088	*
Habitat_destruction_W-Biol_Invasions_L	2518.5	471.5088	*
Habitat_destruction_W-Pollution_L	683	471.5088	*
Habitat_destruction_W-Habitat_destruction_L	1593	471.5088	*
Habitat_destruction_W-Agriculture_L	2699	471.5088	*
Habitat_destruction_W-Overexploitation_NR_L	2105.5	471.5088	*
Agriculture_W-Overexploitation_NR_W	944.5	471.5088	*
Agriculture_W-Flooding_L	1513	471.5088	*
Agriculture_W-Climate_change_L	817	471.5088	*
Agriculture_W-Biol_Invasions_L	1793.5	471.5088	*
Agriculture_W-Pollution_L	42	471.5088	NS
Agriculture_W-Habitat_destruction_L	868	471.5088	*
Agriculture_W-Agriculture_L	1974	471.5088	*
Agriculture_W-Overexploitation_NR_L	1380.5	471.5088	*
Overexploitation_NR_W-Flooding_L	2457.5	471.5088	*
Overexploitation_NR_W-Climate_change_L	1761.5	471.5088	*
Overexploitation_NR_W-Biol_Invasions_L	2738	471.5088	*
Overexploitation_NR_W-Pollution_L	902.5	471.5088	*
Overexploitation_NR_W-Habitat_destruction_L	1812.5	471.5088	*
Overexploitation_NR_W-Agriculture_L	2918.5	471.5088	*
Overexploitation_NR_W-Overexploitation_NR_L	2325	471.5088	*
Flooding_L-Climate_change_L	696	471.5088	*
Flooding_L-Biol_Invasions_L	280.5	471.5088	NS
Flooding_L-Pollution_L	1555	471.5088	*
Flooding_L-Habitat_destruction_L	645	471.5088	*
Flooding_L-Agriculture_L	461	471.5088	NS
Flooding_L-Overexploitation_NR_L	132.5	471.5088	NS
Climate_change_L-Biol_Invasions_L	976.5	471.5088	*
Climate_change_L-Pollution_L	859	471.5088	*
Climate_change_L-Habitat_destruction_L	51	471.5088	NS
Climate_change_L-Agriculture_L	1157	471.5088	*
Climate_change_L-Overexploitation_NR_L	563.5	471.5088	*
Biol_Invasions_L-Pollution_L	1835.5	471.5088	*
Biol_Invasions_L-Habitat_destruction_L	925.5	471.5088	*
Biol_Invasions_L-Agriculture_L	180.5	471.5088	NS
Biol_Invasions_L-Overexploitation_NR_L	413	471.5088	NS
Pollution_L-Habitat_destruction_L	910	471.5088	*
Pollution_L-Agriculture_L	2016	471.5088	*
Pollution_L-Overexploitation_NR_L	1422.5	471.5088	*
Habitat_destruction_L-Agriculture_L	1106	471.5088	*
Habitat_destruction_L-Overexploitation_NR_L	512.5	471.5088	*

Agriculture_L-Overexploitation_NR_L	593.5	471.5088	*

W = world (global), L = local, NR = Natural Resources, Biol = Biological

Asterisks (*) in ***Difference*** column means significant differences (P<0.05).

Friedman's Test. Adjusted for ties Critical Value: 2098.541

P.Value Chisq: 0. F Value: 231.3097. *P*.Value F: 0 .

Post Hoc Analysis Alpha: 0.05 ; df Error: 6903. *t*-Student: 1.960308

Table S3. Models selection for questions Q16 and Q17 (Appendix S1). Selected model use to graphed Figure 4 highlighted in red.

**Cockspur coral tree (*Erythrina crista-galli*, *ceibo* in Spanish) PHOTO**					
**Model**	**Family**	**df**	**logLik**	**AIC**	**Delta**
**~A*NST**	**binomial(logit)**	**4**	**-255.949**	**519.974**	**0.000**
~A+NST	binomial(logit)	3	-258.340	522.726	2.752
~A	binomial(logit)	2	-260.803	525.629	5.655
~NST	binomial(logit)	2	-261.765	527.553	7.579
~NGEO	binomial(logit)	2	-262.452	528.927	1.374
~1 (null)	binomial(logit)	1	-264.262	530.531	10.557
~EL	binomial(logit)	6	-260.062	532.284	12.310
**Cockspur coral tree (*Erythrina crista-galli*, *ceibo* in Spanish) WRITTEN NAME**					
**Model**	**Family**	**df**	**logLik**	**AIC**	**Delta**
**~A+NST**	**binomial(logit)**	**3**	**-125.707**	**257.460**	**0.000**
~A	binomial(logit)	2	-126.846	257.716	0.256
~A*NST	binomial(logit)	4	-125.490	259.055	1.595
~EL	binomial(logit)	2	-127.526	259.076	1.616
~NST	binomial(logit)	2	-127.874	259.770	2.310
~1 (null)	binomial(logit)	1	-129.068	260.143	2.683
~NGEO	binomial(logit)	2	-128.332	260.686	3.226
**Chinaberry tree (*Melia azedarach*, *paraíso* in Spanish) PHOTO**					
**Model**	**Family**	**df**	**logLik**	**AIC**	**Delta**
~NST	binomial(logit)	2	-312.001	628.025	0.000
**~A+NST**	**binomial(logit)**	**3**	**-311.786**	**629.617**	**1.592**
~A*NST	binomial(logit)	4	-311.737	631.549	1.932
~1 (null)	binomial(logit)	1	-315.489	632.985	4.961
~NGEO	binomial(logit)	2	-314.983	633.989	5.965
~A	binomial(logit)	2	-315.273	634.569	6.544
~EL	binomial(logit)	6	-311.977	636.114	8.090
**Chinaberry tree (*Melia azedarach*, *paraíso* in Spanish) WRITTEN NAME**					
**Model**	**Family**	**df**	**logLik**	**AIC**	**Delta**
~NST	binomial(logit)	2	-353.485	710.993	0.000

**~A+NST**	**binomial(logit)**	**3**	**-353.485**	**713.016**	**2.023**
~NGEO	binomial(logit)	2	-355.326	714.674	3.681
~A*NST	binomial(logit)	4	-353.480	715.036	4.043
~1 (null)	binomial(logit)	1	-358.123	718.254	7.261
~A	binomial(logit)	2	-358.123	720.269	9.276
~EL	binomial(logit)	6	-354.379	720.919	9.926
**Water primrose (*Ludwigia grandiflora* subsp. *hexapetala*, *duraznillo de agua* in Spanish) PHOTO**					
**Model**	**Family**	**df**	**logLik**	**AIC**	**Delta**
~NGEO	binomial(logit)	2	-332.569	669.160	0.000
**~A*NST**	**binomial(logit)**	**4**	**-332.245**	**672.566**	**3.405**
~1 (null)	binomial(logit)	1	-335.542	673.092	3.931
~A	binomial(logit)	2	-335.514	675.051	5.891
~NST	binomial(logit)	2	-335.522	675.067	5.907
~EL	binomial(logit)	6	-332.295	676.750	7.590
~A+NST	binomial(logit)	3	-335.494	677.034	7.874
**Water primrose (*Ludwigia grandiflora* subsp. *hexapetala*, *duraznillo de agua* in Spanish) WRITTEN NAME**					
**Model**	**Family**	**df**	**logLik**	**AIC**	**Delta**
**~A+NST**	**binomial(logit)**	**3**	**-325.478**	**657.001**	**0.000**
~NST	binomial(logit)	2	-327.282	658.586	1.585
~A*NST	binomial(logit)	4	-325.470	659.017	2.015
~NGEO	binomial(logit)	2	-331.734	667.491	10.490
~A	binomial(logit)	2	-333.081	670.185	13.184
~1 (null)	binomial(logit)	1	-334.808	671.623	14.622
~EL	binomial(logit)	6	-332.963	678.085	21.084
**Yellow Flag Iris (*Iris pseudacorus*, *lirio amarillo* in Spanish) PHOTO**					
**Model**	**Family**	**df**	**logLik**	**AIC**	**Delta**
**~A+NST**	**binomial(logit)**	**3**	**-325.478**	**657.001**	**0.000**
~NST	binomial(logit)	2	-327.282	658.586	9.038
~A*NST	binomial(logit)	4	-325.470	659.017	9.848
~NGEO	binomial(logit)	2	-331.734	667.491	9.968
~A	binomial(logit)	2	-333.081	670.185	10.027
~1 (null)	binomial(logit)	1	-334.808	671.623	10.766
~EL	binomial(logit)	6	-332.963	678.085	11.870
**Yellow Flag Iris (*Iris pseudacorus*, *lirio amarillo* in Spanish) WRITTEN NAME**					
**Model**	**Family**	**df**	**logLik**	**AIC**	**Delta**
~1 (null)	binomial(logit)	1	-330.221	662.449	0.000
~NST	binomial(logit)	2	-329.770	663.562	1.113
~NGEO	binomial(logit)	2	-329.849	663.721	1.272
~A	binomial(logit)	2	-330.009	664.040	1.592
~EL	binomial(logit)	6	-326.108	664.375	1.927
**~A+NST**	**binomial(logit)**	**3**	**-329.558**	**665.162**	**2.713**
~A*NST	binomial(logit)	4	-328.786	665.648	3.199
**Capybara (*Hydrochoerus hydrochaeris*, *carpincho* in Spanish)**					

**PHOTO**					
**Model**	**Family**	**df**	**logLik**	**AICc**	**Delta**
~NST	binomial(logit)	2	-243.037	490.097	0.000
**~A+NST**	**binomial(logit)**	**3**	**-242.570**	**491.185**	**1.088**
~A*NST	binomial(logit)	4	-242.273	492.622	2.526
~1 (null)	binomial(logit)	1	-246.547	495.102	5.006
~NGEO	binomial(logit)	2	-245.608	495.238	5.142
~A	binomial(logit)	2	-246.074	496.171	6.074
~EL	binomial(logit)	6	-242.482	497.124	7.027
**Capybara (*Hydrochoerus hydrochaeris*, *carpincho* in Spanish) WRITTEN NAME**					
**Model**	**Family**	**df**	**logLik**	**AIC**	**Delta**
**~A+NST**	**binomial(logit)**	**3**	**-184.486**	**375.017**	**0.000**
~A	binomial(logit)	2	-185.505	375.033	0.016
~NGEO	binomial(logit)	2	-185.996	376.014	0.998
~NST	binomial(logit)	2	-186.370	376.763	1.746
~1 (null)	binomial(logit)	1	-187.420	376.847	1.830
~A*NST	binomial(logit)	4	-184.472	377.021	2.004
~EL	binomial(logit)	6	-184.424	381.009	5.992
**Beaver (*Castor canadiensis*, *castor* in Spanish) PHOTO**					
**Model**	**Family**	**df**	**logLik**	**AIC**	**Delta**
~NST	binomial(logit)	2	-314.851	633.725	0.000
**~A+NST**	**binomial(logit)**	**3**	**-314.314**	**634.673**	**0.948**
~1 (null)	binomial(logit)	1	-316.428	634.864	1.139
~NGEO	binomial(logit)	2	-315.882	635.787	2.061
~A	binomial(logit)	2	-315.890	635.802	2.077
~A*NST	binomial(logit)	4	-314.063	636.202	2.476
~EL	binomial(logit)	6	-314.774	641.707	7.982
**Beaver (*Castor canadiensis*, *castor* in Spanish) WRITTEN NAME**					
**Model**	**Family**	**df**	**logLik**	**AIC**	**Delta**
~NST	binomial(logit)	2	-204.577	413.177	0.000
~NGEO	binomial(logit)	2	-204.751	413.524	0.347
**~A+NST**	**binomial(logit)**	**3**	**-204.564**	**415.172**	**1.995**
~A*NST	binomial(logit)	4	-203.930	415.936	2.758
~1 (null)	binomial(logit)	1	-207.149	416.305	3.128
~A	binomial(logit)	2	-207.134	418.292	5.114
~EL	binomial(logit)	6	-204.873	421.906	8.729
**Thrush (*Turdus rufuventris*, *zorzal* in Spanish) PHOTO**					
**Model**	**Family**	**df**	**logLik**	**AIC**	**Delta**
~NST	binomial(logit)	2	-334.882	673.787	0.000
**~A+NST**	**binomial(logit)**	**3**	**-334.558**	**675.162**	**1.375**
~A*NST	binomial(logit)	4	-334.520	677.116	3.329
~1 (null)	binomial(logit)	1	-339.086	680.179	6.392
~A	binomial(logit)	2	-338.765	681.552	7.765
~NGEO	binomial(logit)	2	-338.766	681.554	7.767

~EL	binomial(logit)	6	-335.214	682.589	8.802
**Thrush (*Turdus rufuventris*, *zorzal* in Spanish) WRITTEN NAME**					
**Model**	**Family**	**df**	**logLik**	**AIC**	**Delta**
~NST	binomial(logit)	2	-246.773	497.568	0.000
~1 (null)	binomial(logit)	1	-248.093	498.193	0.625
**~A+NST**	**binomial(logit)**	**3**	**-246.106**	**498.257**	**0.689**
A	binomial(logit)	2	-247.420	498.863	1.295
~A*NST	binomial(logit)	4	-245.989	500.053	2.485
~NGEO	binomial(logit)	2	-248.088	500.198	2.630
~EL	binomial(logit)	6	-244.530	501.219	3.651
**Starling (*Sturnus vulgaris*, *estornino* in Spanish) PHOTO**					
**Model**	**Family**	**df**	**logLik**	**AIC**	**Delta**
~A	binomial(logit)	2	-340.483	684.989	0.000
~1 (null)	binomial(logit)	1	-341.769	685.545	0.555
**~A+NST**	**binomial(logit)**	**3**	**-340.076**	**686.198**	**1.208**
~NST	binomial(logit)	2	-341.360	686.742	1.753
~NGEO	binomial(logit)	2	-341.528	687.078	2.088
~EL	binomial(logit)	6	-337.981	688.122	3.133
~A*NST	binomial(logit)	4	-340.074	688.224	3.235
**Starling (*Sturnus vulgaris*, *estornino* in Spanish) WRITTEN NAME**					
**Model**	**Family**	**df**	**logLik**	**AIC**	**Delta**
~1 (null)	binomial(logit)	1	-228.662	459.331	0.000
~NST	binomial(logit)	2	-227.760	459.542	0.211
~NGEO	binomial(logit)	2	-228.535	461.093	1.762
~A	binomial(logit)	2	-228.658	461.339	2.008
**~A+NST**	**binomial(logit)**	**3**	**-227.756**	**461.557**	**2.226**
~A*NST	binomial(logit)	4	-227.620	463.315	3.984
~EL	binomial(logit)	6	-227.233	466.626	7.294

A = Age, NST = Natural Science Teacher Condition, EL = Education Level, NGEO = Non-governmental Environment Organization Member, * = interaction

Figure S3. Responses from school community members to the question 18 about their knowledge of biological invasions.

Pearson's Chi-squared test

X-squared = 38.812, df = 2, *P*-value = 3.733e-09

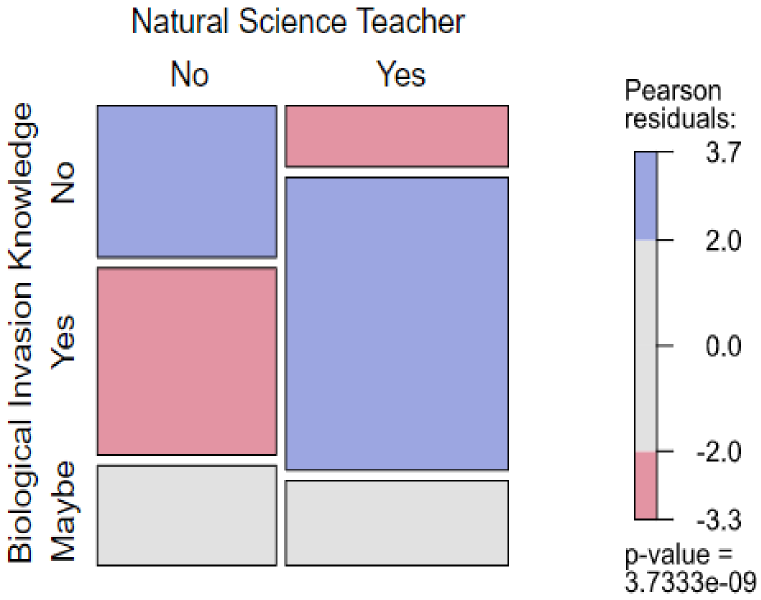

Figure S4. World cloud from NST about their comments on biological invasions. Words are displayed in a word cloud where bigger letter size implies more frequency of presence in replies.

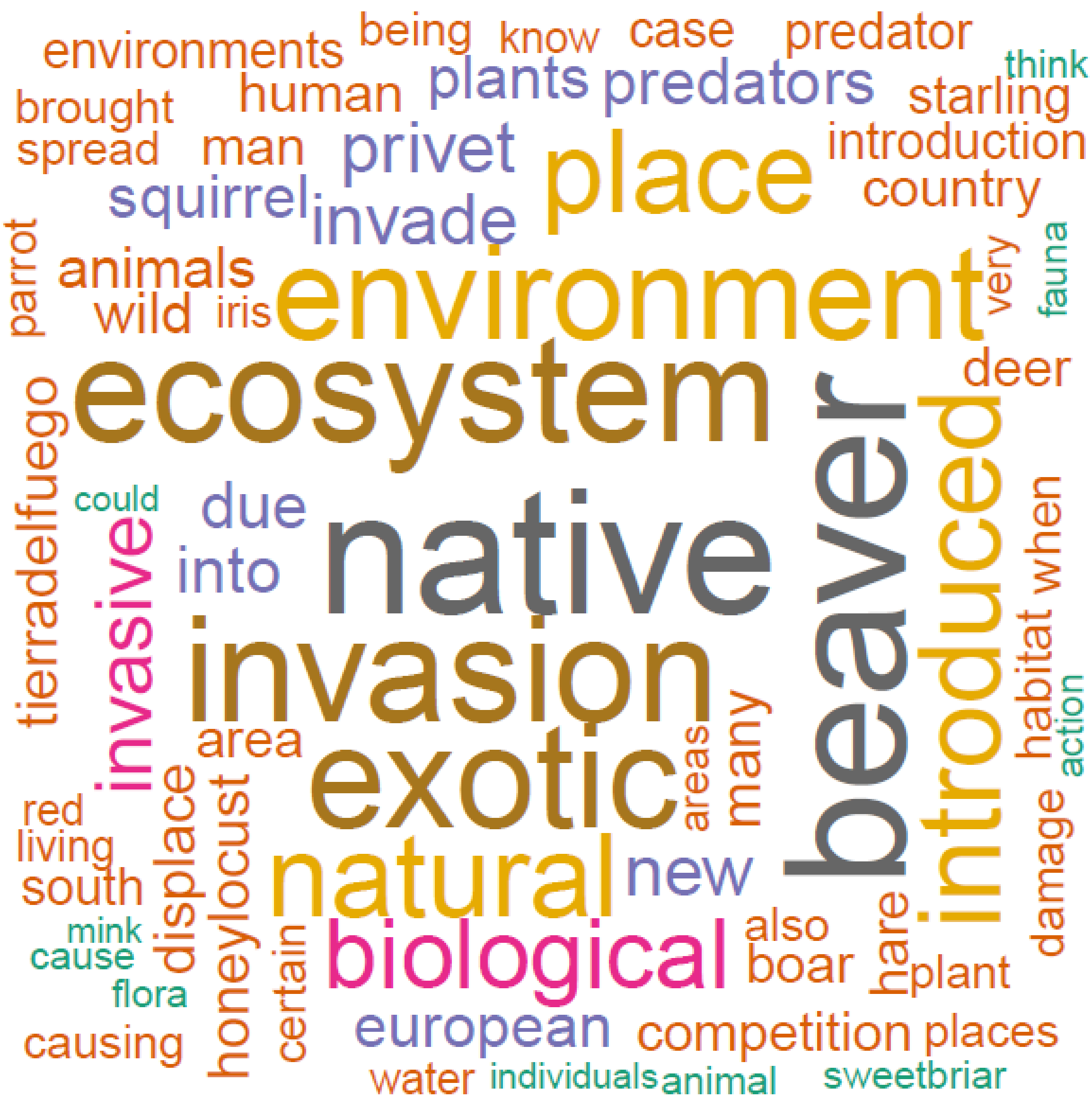

Figure S5. World cloud from non-NST about their comments on biological invasions. Words are displayed in a word cloud where bigger letter size implies more frequency of presence in replies.

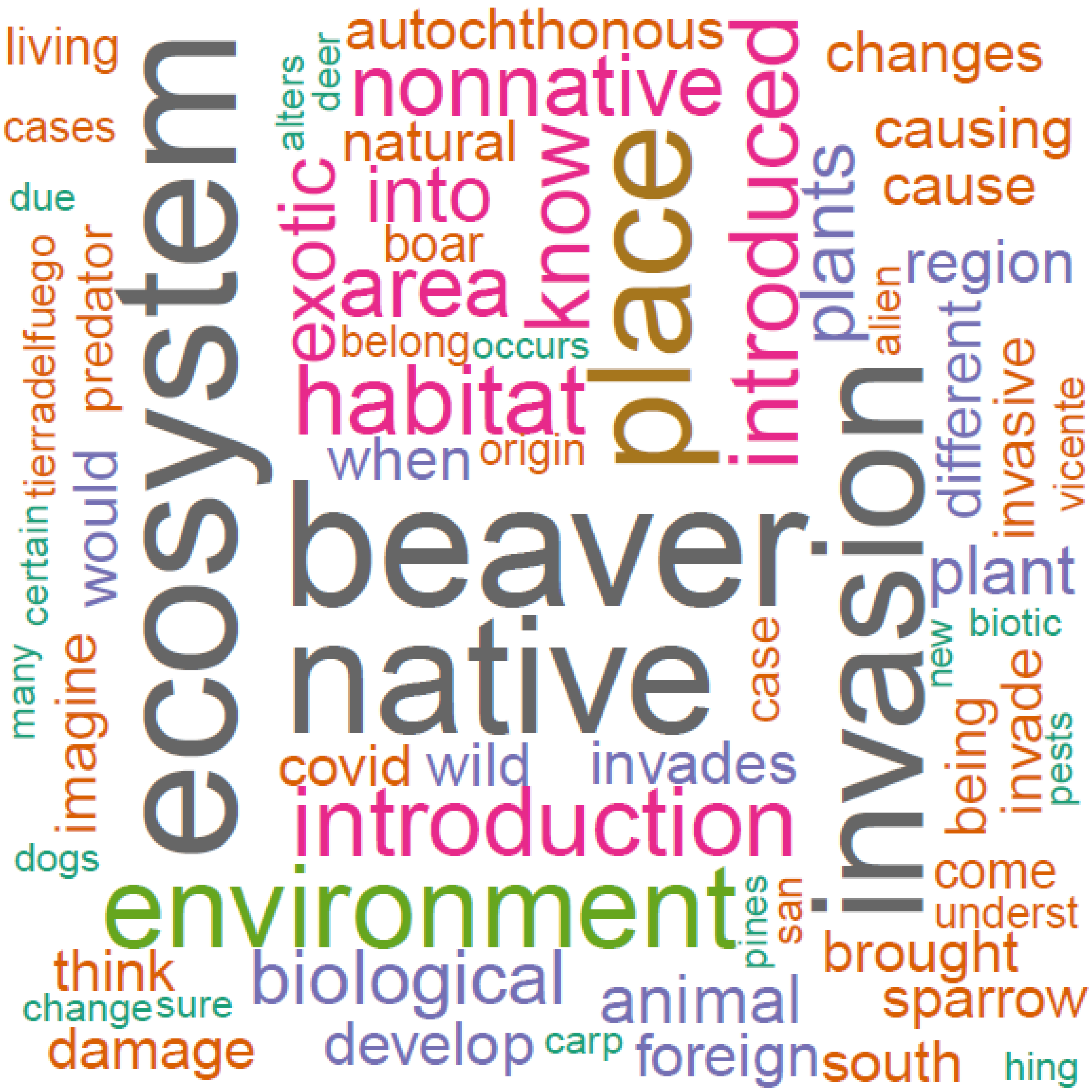

Appendix S3. Preliminary and complementary studies on species recognition

To study the teacher's perception on native and non-native species, we conducted two virtual workshops during the 2020 pandemic. The first one was conducted on October 2nd at *Plaza Ciencia* at San Justo city in Buenos Aires Province, a science fair for teachers and students of all educational levels and the general public. The second one was a workshop organized by the authors at Hurlingham University (http://www.unahur.edu.ar/es/inscripcion-jornadas-y-talleres-de-integracion-y-articulacion-escuela-universidad). The attendees were primarily teachers and student teachers from several parts of the country. As part of the second workshop, people were divided into two virtual rooms. Attendees were randomly assigned to one of each room with different topics boarded using a random tool in Zoom. We wanted to know how people considered native and non-native species, so they were asked if the species *CEIBO* (Spanish name of Cockspur coral tree, *Erythrina crista-galli*) was a native or non-native species. One room only saw the written name of the species in Spanish and the scientific name, and the other room only saw a photograph of the plant (an image of the whole tree and detail of flowers).

Perception of attendees was analysed using a Chi-square test for frequency of responses.

As a result, most people recognized the species as a native one, but such selection depended on if they saw the written name or the photograph. More people considered the species as native when it was presented as a written word instead of a photograph.

Table S4. Responses (%) to the native consideration of the *CEIBO*, national flower of Argentina, to attendees of the workshop.

	**Photograph (%)**	**Written name (%)**
**Native consideration**	47 (77.1)	56 (91.8)
**Non-native consideration**	14 (22.9)	5 (8.2)
Marginal Column Totals	61	61

The chi-square statistic is 5.0496. The *P*-value is 0.024632. Significant at $P<0.05$.

The chi-square statistic with Yates correction is 3.9898. The *P*-value is 0.045777. Significant at $P<0.05$.
